# Approaching psychotherapy for people from international buddhist organisations

**DOI:** 10.1192/j.eurpsy.2021.1334

**Published:** 2021-08-13

**Authors:** A.I.M. Anders

**Affiliations:** Institute Of Social And Cultural Anthropology, Ludwig-Maximilians-University Munich, Munich, Germany

**Keywords:** Buddhism, crazy wisdom, karma purification, abuse

## Abstract

**Introduction:**

Mindfulness techniques, which are currently widely used in psychosomatics and psychotherapy, pose challenges when treating people coming from Buddhist groups for several reasons.

**Objectives:**

For their treatment, it is important to take into account decontextualized terms that underlie crucial group dynamics and the effects of damaging neologisms in international Buddhist organizations.

**Methods:**

In the current research project, this topic is approached in combining quantitative with qualitative data. Whereas the data collection is still ongoing, the replies of twelve people are presented.

**Results:**

As commitments to secrecy hinder people to ask for psychotherapy for long, they were asked on their thoughts about secrecy in Buddhist groups. Five of them agreed that acts against them were declared secret, which they then further specified. Six probands agreed having witnessed acts directed toward others being sworn to secrecy, four of which told this was about sexual abuse. Whereas nine agreed having experienced enemy images being built up, three agreed and specified how their own freedom was impaired and six witnessed and specified other group members’ freedom having been constrained. While six persons agreed that it was assumed in their group one or more persons could ‘purify’ someone else in the sense of a ‘karma purification’ and specified their replies, two replied this concept was used to rationalize actions towards themselves and how it has affected.

**Conclusions:**

As for psychotherapy, it is important to take into account rationalization of violence and abuse through neologisms, pseudotherapies and structural issues in context.

**Conflict of interest:**

This research is funded by the German Federal Ministry of Education and Research (BMBF).

